# Administration practice and adherence of nusinersen in adults with 5q-spinal muscular atrophy in China: an ambispective multicenter study

**DOI:** 10.1186/s12883-026-04730-x

**Published:** 2026-02-28

**Authors:** Xiaoli Yao, Yi Dai, Wenhua Zhu, Yuying Zhao, Huifang Shang, Qiang Meng, Yaling Liu, Liqiang Yu, Daojun Hong, Juanjuan Chen, Lu Shen, Wanjin Chen, Ken Chen, Li Zhang, Maerhaba Mai, Liying Cui

**Affiliations:** 1 Department of Neurology, The First Affiliated Hospital, Sun Yat-sen University; Guangdong Provincial Key Laboratory of Diagnosis and Treatment of Major Neurological Diseases, National Key Clinical Department and Key Discipline of Neurology, Guangzhou, China; 2 Department of Neurology, Peking Union Medical College Hospital, Chinese Academy of Medical Sciences, Beijing, China; 3 Department of Neurology, Huashan Hospital, Fudan University, Shanghai, China; 4 Department of Neurology, Qilu Hospital of Shandong University, Jinan, China; 5 Department of Neurology, West China Hospital, Sichuan University, Chengdu, China; 6 Department of Neurology, The First People’s Hospital of Yunnan Province, Kunming, China; 7 Department of Neurology, The Second Hospital of Hebei Medical University, Shijiazhuang, China; 8 Department of Neurology, The First Affiliated Hospital of Soochow University, Suzhou, China; 9 Department of Neurology, The First Affiliated Hospital of Nanchang University, Nanchang, China; 10 Department of Neurology, Peking University Shenzhen Hospital, Shenzhen, China; 11 Department of Neurology, Xiangya Hospital of Central South University, Changsha, China; 12 Department of Neurology, The First Affiliated Hospital of Fujian Medical University, Fuzhou, China; 13 Real World Solutions, IQVIA Solutions Enterprise Management Consulting (Shanghai) Co., Ltd, Shanghai, China; 14 Medical Department, Biogen Biotechnology (Shanghai) Co., Ltd, Shanghai, China

**Keywords:** Nusinersen, Adult, Registry, Adherence, Administration practice

## Abstract

**Background:**

Due to nusinersen’s route of administration and dosing regimen, the spinal muscular atrophy (SMA) population and/or their caregivers need to travel to hospitals and work around physician’s schedules to receive treatment. Real-world studies with large sample sizes on administration practice and adherence rate of nusinersen are lacking, especially in non-United States settings. This study aimed to investigate the administration practice and adherence of nusinersen in Chinese adults with 5q-SMA.

**Methods:**

An ambispective, multicenter registry of adults with 5q-SMA in China provided the longitudinal data for this analysis. Nusinersen was scheduled on Day 0, 14, 28, 63, and every 4 months thereafter. Adherence rate was calculated at dose level. A dose was deemed adherent if the interval between the current and preceding dose aligned with the standard dosing regimen, allowing a grace period of ± 7 days for doses 2 through 4 and ± 28 days for all subsequent doses.

**Results:**

A total of 177 participants receiving nusinersen with 1,329 doses were included in the study. All injections were given in inpatient setting with no ventilatory support or sedation needed. Only one injection (0.1%) was performed under general anesthesia. Almost all (*n* = 1,322, 99.5%) injections were given through interspinous lumbar puncture. Injections in participants with scoliosis require more imaging techniques for guiding administration compared with those in participants without scoliosis (68.3% vs. 29.1%). Ultrasound was the most commonly used guide before or during administration (674/708). The adherence rate was 92.8% (1,062/1,144). The median inter-dose intervals aligned well with dosing schedule, with 14 days for Dose 2 and 3, 35 days for Dose 4, and 120–124 days for Dose 5 to 10 as maintenance doses.

**Conclusions:**

Findings characterize the patterns of nusinersen administration practice, demonstrating high real-world adherence to nusinersen administration in Chinese adults with 5q-SMA.

**Trial registration:**

The registry was registered at clinicaltrials.gov (NCT05618379) on November 16^th^, 2022.

**Supplementary Information:**

The online version contains supplementary material available at 10.1186/s12883-026-04730-x.

## Background

5q-spinal muscular atrophy (SMA) is a neuromuscular disorder caused by a homozygous deletion or mutation in the *survival motor neuron 1* (*SMN1*) gene on chromosome 5q [31]. The resulting SMN protein deficiency leads to the degeneration and dysfunction of motor neurons in the spinal cord. SMA is characterized by a broad spectrum of clinical presentations, including muscle atrophy, muscle weakness, progressive loss of motor function, scoliosis, and often early mortality [7, 48] Subtypes of SMA are classified based on the age at disease onset and the best motor milestone achieved, ranging from the most severe infantile form, SMA Type I, to the least severe adult onset form, SMA Type IV [36]. SMA Type II-IV primary comprise the adult forms. Due to varying disease types and duration since symptom onset, adults with SMA may vary significantly in gross motor function, ranging from non-sitters to walkers.

Nusinersen is an antisense oligonucleotide that modifies the splicing of *SMN2* precursor messenger ribonucleic acid (mRNA) increasing full-length SMN protein levels [27]. As the first approved disease-modifying therapy (DMT) for 5q-SMA, nusinersen has significantly transformed the standard of care for affected individuals. Extensive clinical trials and observational studies demonstrate that nusinersen improves survival and motor function while maintaining a favorable safety profile in adults [14, 15, 20, 41, 44].

Nusinersen is intended exclusively for intrathecal administration, with each dose (12 mg/5 mL) delivered into the subarachnoid space to maximize drug concentration in the central nervous system. The 12/12 mg nusinersen dosing schedule comprises four loading doses administered over the initial two months, followed by maintenance doses every four months thereafter [28, 10]. Alternative dosing regimens of nusinersen, with high dosage (28 mg and 50 mg) and fewer loading doses, have been approved by European Union and Japan [10, 33]. Sedation or anesthesia may be required based on the adults’ clinical condition. Ultrasound or other imaging modalities can be employed to guide administration, particularly in individuals with pronounced spinal deformities [39]. Previous studies have reported a consistently high technical success rate for nusinersen administration using conventional lumbar puncture (LP) [1, 40, 45]. In certain cases, such as in individuals with scoliosis or a history of spinal surgery, anatomical complexity may hinder conventional LP, necessitating alternative techniques and/or imaging guidance [11, 16, 22, 25].

Given nusinersen’s administration route and dosing schedule, individuals with SMA and/or their caregivers must travel to healthcare facilities and coordinate with physicians’ availability to receive treatment [24, 49]. Prior observational studies utilizing claims databases from the United States (US) demonstrated varying adherence rates to nusinersen, ranging from 30.0% to 80.5%, [12, 13, 46] but it is important to note that the inclusion/exclusion criteria used in these studies were likely insufficient to identify the individuals with complete nusinersen dosing history. Studies using data from electronic health records (EHRs) in the US suggested higher adherence rates at 92.0% and 93.9%, respectively [9, 46]. These studies had varying sample sizes (number of doses ranging from 93 to 1,462). Real-world evidence on nusinersen administration practices and adherence rates remains limited, particularly outside the US.

To address this gap, a national SMA registry focusing on Chinese adults with 5q-SMA was established in 2023 [6]. This analysis aims to assess real-world administration practices and adherence to nusinersen among adults in China using data from this registry.ai 

## Methods

### Study design and setting

In 2023, a multicenter, longitudinal registry was initiated in China to retrospectively and prospectively collect clinical data on adults with 5q-SMA [6]. The registry included 12 general and specialized hospitals nationwide, with recruitment commencing in January 2023 (See Additional File 1 for the hospital list). The first interim analysis utilized registry data to capture participants’ baseline characteristics, along with detailed records of nusinersen dosing and administration. The index date was defined as the initiation of nusinersen therapy, with baseline data collected within 60 days prior. The study period spanned from April 28^th^, 2019—the date of nusinersen’s launch in China—through January 26^th^, 2024, the data cut-off date for this analysis.

### Participants

To be eligible for inclusion in this registry, participants must meet the following criteria: (1) Ability of the participant and/or his/her legally authorized representative to understand the purpose and risks of the study, to provide informed consent, and to authorize the use of confidential health information in accordance with national and local privacy regulations; (2) Genetically confirmed 5q-SMA; (3) Adults (age ≥ 18 years) at registry enrollment.

Participants starting nusinersen treatment during the study period, regardless of the duration of treatment, dosing frequency, or whether other DMT was used prior to, together with or after nusinersen treatment, were included in this analysis.

### Variables

Baseline demographic and clinical characteristics were collected and evaluated among participants with known first-dose record, including age, sex, SMA type, diagnosis of scoliosis, and number of doses administered. Nusinersen doses and administration details at each dose, including date of administration, care setting of administration (inpatient or outpatient), ventilatory support during administration, method of administration, use of sedation and general anesthesia, and imaging guidance used were also collected and evaluated among all participants with nusinersen use.

Adherence rate was calculated in participants with known first-dose record and with two or more doses. The anticipated dosing schedule followed the package insert, comprising loading doses on days 0, 14, 28, and 63, followed by maintenance doses at four-month intervals [28]. A dose was deemed adherent if the interval between the current and preceding dose aligned with the standard dosing regimen, allowing a grace period of ± 7 days for doses 2 through 4 and ± 28 days for all subsequent doses. Dose-level adherence was defined as the proportion of doses administered on schedule relative to the total number of doses, where Dose 1 was excluded from the calculation. The definition of dose-level adherence rate was based on the recommendations for delayed or missed dose per package insert, which suggested administering the dose immediately followed by the treatments per scheduled inter-dose intervals.

### Statistical methods

Descriptive analyses were used to display the demographic and clinical characteristics of participants and records of dosing details overall, by gross motor function and by scoliosis diagnosis. Continuous variables were summarized using the arithmetic mean and standard deviation (SD) and/or the median, first quartile (Q1) and third quartile (Q3). Categorical variables were presented as counts and percentages by category. A box plot was generated to illustrate the distribution of dosing intervals for each dose. All analyses were performed using SAS^®^ (SAS Institute Inc., Cary, NC; version 9.2 or later) and R Statistical Software (v 4.3.3; R Core Team 2023).

## Results

### Baseline characteristics and nusinersen doses of participants

As of January 26^th^, 2024, a total of 200 participants were screened and enrolled in the registry. Of them, 177 participants used nusinersen with a total of 1,329 doses, thus were included in this analysis. Among 177 participants, 174 participants (98.3%) had known first-dose record, of whom 172 participants (98.9%) used nusinersen as their first DMT. The baseline characteristics of the 172 participants are shown in Table 1. The median (Q1, Q3) number of nusinersen doses administered per participants was 8 (7, 9) among participants with known first-dose record, 8 (7, 8) among non-sitters, 8 (7, 9) among sitters, and 8 (6, 9) among walkers.

**Table 1:** Baseline characteristic among participants with known first-dose record and using nusinersen as their first DMT

	Total*	Non-sitter	Sitter	Walker
Number of participants	172	38	58	75
Age at nusinersen initiation (years)				
Median (Q1, Q3)	26 (14, 52)	27 (21, 33)	26 (19, 33)	25 (20, 34)
Sex, n (%)				
Male	104 (60.5%)	17 (44.7%)	34 (58.6%)	52 (69.3%)
Female	68 (39.5%)	21 (55.3%)	24 (41.4%)	23 (30.7%)
SMA type, n (%)				
1	1 (0.6%)	0 (0.0%)	1 (1.7%)	0 (0.0%)
2	37 (21.5%)	27 (71.1%)	10 (17.2%)	0 (0.0%)
3	131 (76.2%)	11 (28.9%)	47 (81.0%)	72 (96.0%)
4	3 (1.7%)	0 (0.0%)	0 (0.0%)	3 (4.0%)
Diagnosis of scoliosis, n (%)				
Yes	107 (62.2%)	33 (86.8%)	47 (81.0%)	27 (36.0%)
No	65 (37.8%)	5 (13.2%)	11 (19.0%)	48 (64.0%)

*DMT* Disease Modifying Therapy, *Q1* First Quartile, *Q3* Third Quartile, *SMA* Spinal Muscular Atrophy

* Of the 172 total participants, one participant’s gross motor function was unknown at baseline

### Administration practice of nusinersen

Of the 1,329 injections during the analysis period (Table 2), all injections were given in inpatient setting with no ventilatory support or sedation needed. Only one injection (0.1%) was performed under general anesthesia. Almost all (*n* = 1,322, 99.5%) injections were given through interspinous LP. Other administration methods included Ommaya capsular sheath (*n* = 6, 0.5%) and dural puncture (*n* = 1, 0.1%). Notably, the dural puncture and general anesthesia came from one single nusinersen injected during a spine surgery.

**Table 2:** Administration details of Nusinersen injections

	Total	Non-sitter	Sitter	Walker	With scoliosis	Without scoliosis
Number of participants	177*	38	58	75	108	69
Number of injections	1329	285	461	554	820	509
Care setting, n (%)						
Inpatient	1329 (100.0%)	285 (100.0%)	461 (100.0%)	554 (100.0%)	820 (100.0%)	509 (100.0%)
Ventilatory support, n (%)						
Yes	0 (0.0%)	0 (0.0%)	0 (0.0%)	0 (0.0%)	0 (0.0%)	0 (0.0%)
No	1324 (100.0%)	285 (100.0%)	456 (100.0%)	554 (100.0%)	820 (100.0%)	504 (100.0%)
Unknown	5 (0.4%)	0 (0.0%)	5 (1.1%)	0 (0.0%)	0 (0.0%)	5 (1.0%)
Use of anesthesia or sedation, n (%)						
Yes, anesthesia	1 (0.1%)	0 (0.0%)	1 (0.2%)	0 (0.0%)	1 (0.1%)	0 (0.0%)
Yes, sedation	0 (0.0%)	0 (0.0%)	0 (0.0%)	0 (0.0%)	0 (0.0%)	0 (0.0%)
No	1328 (99.9%)	285 (100.0%)	460 (99.8%)	554 (100.0%)	819 (99.9%)	509 (100.0%)
Method of administration, n (%)						
Interspinous lumbar puncture	1322 (99.5%)	279 (97.9%)	460 (99.8%)	554 (100.0%)	813 (99.1%)	509 (100.0%)
Ommaya capsular sheath	6 (0.5%)	6 (2.1%)	0 (0.0%)	0 (0.0%)	6 (0.7%)	0 (0.0%)
Dural puncture	1 (0.1%)	0 (0.0%)	1 (0.2%)	0 (0.0%)	1 (0.1%)	0 (0.0%)
Guide before/during administration, n (%)						
Ultrasound	674 (50.7%)	224 (78.6%)	285 (61.8%)	145 (26.2%)	540 (65.9%)	134 (26.3%)
Fluoroscopy or radioscopy	25 (1.9%)	0 (0.0%)	24 (5.2%)	1 (0.2%)	16 (2.0%)	9 (1.8%)
CT-scan	4 (0.3%)	3 (1.1%)	1 (0.2%)	0 (0.0%)	4 (0.5%)	0 (0.0%)
Unknown	5 (0.4%)	0 (0.0%)	5 (1.1%)	0 (0.0%)	0 (0.0%)	5 (1.0%)
Not using imaging techniques	621 (46.7%)	58 (20.4%)	146 (31.7%)	408 (73.6%)	260 (31.7%)	361 (70.9%)

*CT* Computed Tomography

For percentage calculation in all categorical variables, "unknown" or "missing" is not included in the denominator except for guide before/during administration

* Gross motor function was only analyzed among participants using nusinersen as their first DMT. Of the 177 participants, only 171 participants had known gross motor function status

The percentage of injections that did not utilize any imaging guide prior to or during administration among non-sitters, sitters and walkers was 20.4%, 31.7% and 73.6%, respectively. Injections in participants with scoliosis require more imaging techniques for guiding administration compared with those in participants without scoliosis (68.3% vs. 29.1%). Ultrasound was the most commonly used guide before or during administration (674/708), accounting for 95.2% of imaging-guided methods. Other imaging-guided methods included fluoroscopy or radioscopy, and computed tomography (CT)-scan.

### Adherence to nusinersen

**Fig. 1:**
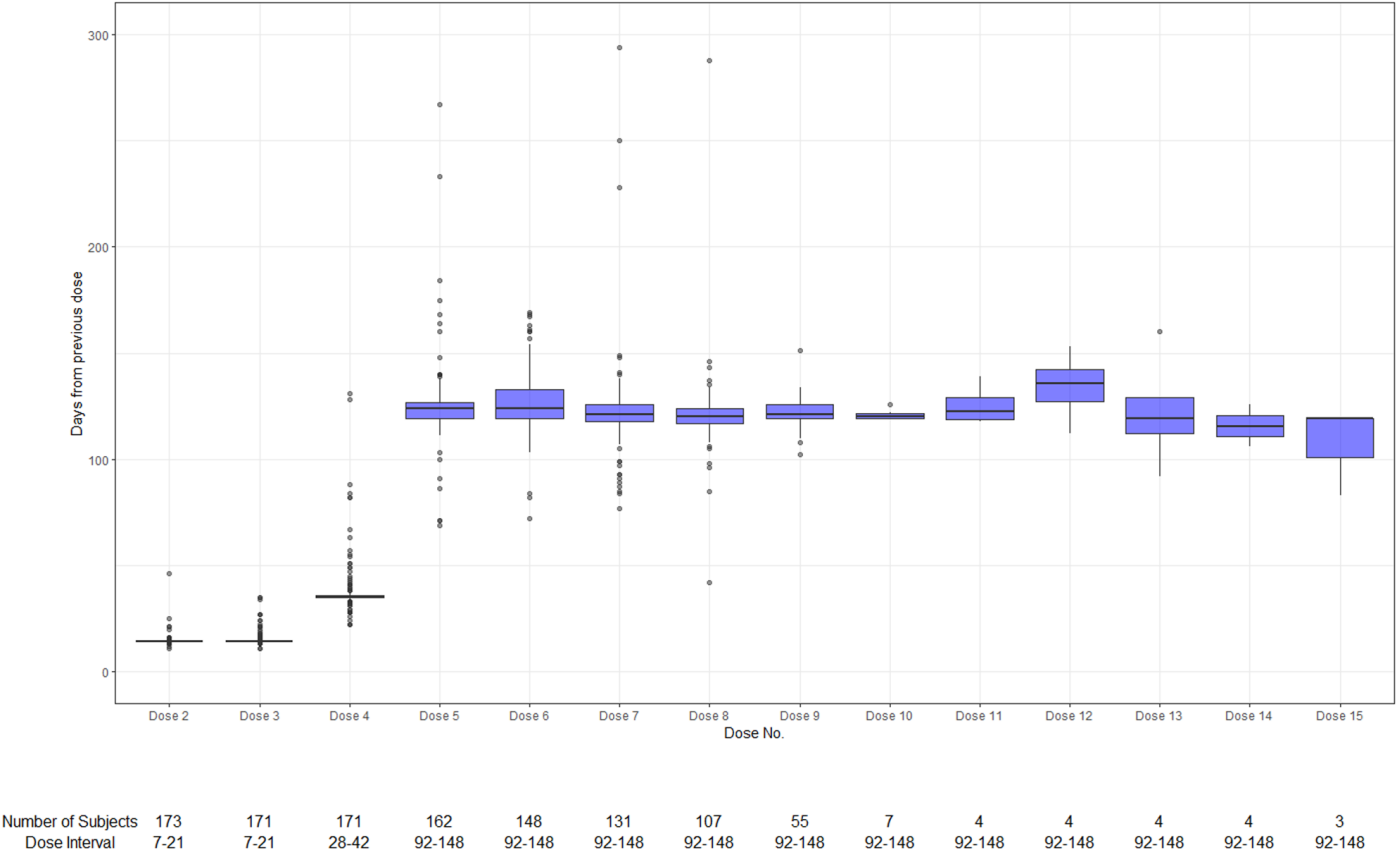
Boxplot of inter-dose interval for nusinersen Note: For Dose 3, one record with 536 days from previous dose is not displayed in the figure

Distribution of inter-dose intervals at each dose is shown in Table 3; Fig. 1. At dose level, the overall adherence rate was 92.8% (1,062/1,144). The median dose intervals aligned well with dosing schedule, with 14 days for Dose 2 and 3, 35 days for Dose 4, and 120–124 days for Dose 5 to 10. Specifically, 4 dose intervals > 240 days were observed during the maintenance phase (2 for Dose 7, and 1 for Dose 5 and 8 each).

**Table 3:** Nusinersen adherence at dose-level by inter-dose interval in days

	Dose 2	Dose 3	Dose 4	Dose 5	Dose 6	Dose 7	Dose 8	Dose 9	Dose 10	Dose 11	Dose 12	Dose 13	Dose 14	Dose 15
Nx	173	171	171	162	148	131	107	55	7	4	4	4	4	3
Mean (SD)	14.4 (2.76)	18.2 (39.99)	38.0 (13.66)	125.3 (19.56)	126.3 (14.85)	123.1 (23.92)	120.9 (19.78)	121.5 (6.85)	120.9 (2.54)	125.5 (9.68)	134.0 (17.07)	122.5 (28.05)	115.8 (8.66)	107.0 (20.78)
Median	14	14	35	124	124	121	120	121	120	122.5	135.5	119	115.5	119
Q1, Q3	14, 14	14, 14	35, 36	119, 127	119, 133	118, 126	117, 124	119, 126	119, 122	118.5, 132.5	122, 146	105.5, 139.5	109, 122.5	83, 119
Min, Max	11, 46	11, 536	22, 131	69, 267	72, 169	77, 294	42, 288	102, 151	119, 126	118, 139	112, 153	92, 160	106, 126	83, 119
Expected Interval	7–21	7–21	28–42	92–148	92–148	92–148	92–148	92–148	92–148	92–148	92–148	92–148	92–148	92–148
Number (%) of doses on time*	171 (98.8%)	161 (94.2%)	147 (86.0%)	150 (92.6%)	132 (89.2%)	121 (92.4%)	104 (97.2%)	54 (98.2%)	7 (100.0%)	4 (100.0%)	2 (50.0%)	3 (75.0%)	4 (100.0%)	2 (66.7%)

*SD* Standard Deviation, *Q1* First Quartile, *Q3* Third Quartile

* Use grace period of ± 7 days for loading doses (Dose 2 to 4) and ± 28 days thereafter from the expected interval

## Discussion

This analysis represents the first investigation into the administration practices of and adherence to nusinersen in adults with 5q-SMA in China. Utilizing data from a comprehensive nationwide registry, the analysis included both prospective and retrospective data, and featured a substantial sample size with detailed records of nusinersen doses. Given that the registry data predominantly originated from EHRs where intrathecal procedures were clearly documented within hospital settings, the registry stands as a dependable resource for examining the intrathecal procedure and medication adherence among our target population. In this analysis, all nusinersen doses were administered in an inpatient setting. This finding contrasts with other studies conducted outside of China, where outpatient nusinersen administration is not uncommon [25, 29, 37, 38]. This discrepancy is likely attributable to the higher reimbursement rates for inpatient versus outpatient medical expenses in China, leading to a general tendency for hospitalization across various medical conditions [23, 43]. Even though nusinersen administration typically does not require advanced supporting technologies, e.g., general anesthesia, imaging guidance, economic factors may still play a crucial role in determining the choice of care setting. The use of general anesthesia or sedation prolongs procedure time and recovery, increases costs, and imposes additional burdens on intrathecal administration. In the current analysis, only one dural puncture required general anesthesia (*n* = 1, 0.1%). We also found that no nusinersen doses were administered with sedation. Previous studies also reported limited use of general anesthesia or sedation among adults with nusinersen, even when using transforaminal approach [1]. Comparably, a Chinese study leveraging an SMA disease registry on a pediatric population reported a similar percentage of general anesthesia use (0.1%) but a much higher percentage of sedation use (9.0%) [32]. Together with previous studies, our findings reflect that utilization of general anesthesia is relatively low across different populations administered with nusinersen, while sedation use may be primarily related to individual cooperation since adults are generally more cooperative during medical procedures compared with children. Also, the peri-procedural pain can be effectively managed with local anesthesia, making general anesthesia and sedation redundant in most cases. The low rates of general anesthesia and sedation observed in our analysis suggest a low burden of potential side effects from general anesthesia and sedation among the adult SMA population, as well as a relatively low burden of healthcare resource utilization caused by general anesthesia or sedation in China. Most of intrathecal nusinersen injections were administered via conventional interspinous LP. Conventional interspinous LP has been shown to be well tolerated in adults with 5q-SMA with a high technical success rate, and it is typically the preferred method for administering nusinersen in this SMA population without contraindications. [1, 40, 45]. In the SMA population with symptomatic scoliosis, osseous fusion, or prior spine surgeries, conventional interspinous LP may pose challenging due to spinal deformities. In such cases, alternative approaches to intrathecal access, e.g., transforaminal LP, cervical puncture, and lumbar laminotomy, may be required [19, 26, 40, 45]. These alternative methods have greater technical difficulty and increased risks of complications compared with conventional interspinous LP [1, 2]. Despite scoliosis accounting for > 60% of participants in the analysis, conventional interspinous LP still dominated the method of administration. This is possibly because all sites in our registry are SMA centers of excellence, and Chinese physicians are experienced in performing conventional interspinous LP, even under the complex spinal deformities, due to high volume of SMA patients they treat. Even though the functional status and degree of scoliosis of adults are quite different in clinical practice, nusinersen administration can still be mostly performed by conventional interspinous LP for most SMA populations in the registry sites. Notably, one participant with scoliosis utilized Ommaya capsular sheath to administer nusinersen. To date, very limited cases of nusinersen administration through an Ommaya reservoir have been reported among SMA adults and children with complex spinal deformities [3, 17, 21, 30]. The use of Ommaya reservoirs is a viable, practical method for repeated infusions with the potential to control administration costs and achieve therapeutic value, although it might be subject to certain complications, e.g., infections and catheter separation. Previous research on nusinersen administration have established that conventional interspinous LP without imaging guidance is feasible and safe for non-complex population, characterized by a Cobb angle of 50 degrees or less and no history of spinal surgery [1, 2]. Although there was a lack of Cobb angle data in this analysis, participants with scoliosis were more likely to require imaging guidance, with 68.3% of injections for participants with scoliosis using imaging-guided injections compared with 29.1% in those without scoliosis. This observation is consistent with previous study indicating that scoliosis complicated LP procedures, often necessitated advanced support to ensure accurate and safe administration [1, 3, 4, 35, 40, 42, 47]. Among imaging-guided methods, ultrasound was the most frequently utilized imaging technique (95.2%) in our analysis. Ultrasound, fluoroscopy and CT scans are choices of imaging-guided intrathecal administration of nusinersen as recommended in the package insert.(Nusinersen Sodium Injection, 2019) Compared with fluoroscopy or CT scans, ultrasound is radiation-free, preventing repeated radiation exposure from long-term nusinersen administrations [18]. As shown in this study, Chinese physicians are confident at using ultrasound for guiding nusinersen administration in most cases to minimize unnecessary harm to participants. These findings suggest the real-world clinical practice of tailoring nusinersen administration practices to individual need, particularly considering the presence of spinal deformities, which aligns with employing a personalized approach to nusinersen administration as suggested by the SMA guideline for adolescents and adults in China [34].

In prior research, adherence rates showed considerable variation, which could be attributed to inconsistent definitions of adherence, diverse data sources, and differences in medical practices across institutions [9, 12]. The registry employed in our analysis, primarily derived from EHRs used in routine clinical care, offers a comprehensive data collection and a less biased assessment of nusinersen adherence. Our analysis revealed a high adherence rate of 92.8% (1,062 out of 1,144 doses) beginning from the second dose in adult participants, closely aligning with the adherence rates observed in a pediatric study conducted in China [32]. This finding is particularly significant considering that the analysis period largely coincided with the coronavirus disease 2019 (COVID-19) pandemic and multiple lockdowns in China, and the obstacles to traveling to hospitals for administration, highlighting the importance the SMA population and their families placed on nusinersen as an essential aspect of disease management. The inclusion of nusinersen in the National Reimbursement Drug List in China significantly improved accessibility and adherence for adults with 5q-SMA [8]. In addition, nusinersen shows notable effectiveness and safety profile among adults with SMA, thus increasing the SMA population’s willingness to be treated on time. The high adherence rates across different doses underscore the effectiveness of using EHR-based registries for monitoring and assessing adherence with nusinersen treatment. It is noteworthy that we observed several extended inter-dose intervals exceeding 240 days (cutoff of necessity to give additional loading dose(s) during the maintenance phase), indicating the resumption of treatment following a dose interruption. With consideration of the timeframe of this study, the reasons of the resumption of treatment might include lockdown lift, dynamic change of reimbursement policy and economic status of participants. Although we did not gather the specific reasons for these extended intervals, the data highlight the variability in nusinersen treatment patterns in real-world practice.

The present analysis has several significant implications for nusinersen treatment in adults with 5q-SMA. First, it addresses a crucial gap in current knowledge by providing comprehensive data on nusinersen administration and adherence among the adult population. With a large sample size of adult participants and nusinersen doses, our findings offer valuable insights into the real-world use of nusinersen. Second, our results can serve as a foundation for developing guidelines and protocols for nusinersen administration across various healthcare settings. For instance, ultrasound should be recommended as the primary method of choice if individuals need imaging-guided intrathecal administration. By establishing standardized practices and institutional protocols, healthcare professionals can ensure consistent and appropriate administration of nusinersen, ultimately enhancing level of care. Third, our results help facilitate and provide adults with SMA and their families with a better understanding and clearer expectation of the administration process. For instance, it is most likely that they should prepare for a hospitalization at each scheduled administration time, and may encounter irregular method of administration if the individual’s spinal situation is more complex. Peri-procedural pain is not uncommon but should mostly be well-managed without general anesthesia. Last, this analysis provides essential background information and insights that can inform future research, including the association between adherence and clinical outcomes, cost-effectiveness of different administration methods, the impact of administration practices on safety events, and the relationship between individual characteristics and administration practices. With the foreseeable introduction of high-dose nusinersen in more countries including China, this analysis will also guide future research regarding adherence to nusinersen therapy across various contexts, encompassing both the high-dose regimen with reduced number of loading doses and the transition from approved 12/12 mg regimen to high-dose nusinersen.

There are several limitations to our analysis that need to be acknowledged. First, the study design may introduce inherent selection bias, given that it relies on a disease registry with retrospective visits. Individuals not able to access nusinersen treatment for various reasons (e.g., skeptical about treatment effectiveness due to long-term disability, with low socioeconomic status) are underrepresented in this registry. The inclusion of adult participants already receiving nusinersen and the need for informed consent upon enrollment may result in a survivor bias, favoring those with a better prognosis and potentially leading to an overestimation of adherence rates. Second, due to the lack of Cobb’s angle data, we were unable to determine the severity of scoliosis and the subsequent association between scoliosis severity and the administration details of injections. Third, the analysis did not account for discontinuation rates of nusinersen, including the specific percentages, dose numbers, and underlying reasons. These factors are critical indicators of non-adherence and warrant further examination. Fourth, this analysis did not evaluate the association between adherence rates and key clinical outcomes, such as improvements in motor function. This limitation reduces the immediate clinical applicability of the findings and highlights the need for future research to explore the impact of adherence on important patient outcomes. Lastly, our findings are based on an interim analysis of the registry data. Consequently, the sample sizes for maintenance doses in the later stages (Dose 10 and beyond) are relatively small due to the limited length of follow-up period. This limitation is expected to be addressed as the registry continues with more long-term data collected.

## Conclusions

This study represents the first comprehensive investigation into the administration practices and real-world adherence to nusinersen in Chinese adults with 5q-SMA. The low burden associated with the administration process, with minimal need for advanced anesthesia or sedation, suggests that nusinersen is generally well-tolerated in this population. Furthermore, the study provides critical insights into the administration methods, particularly in participants with scoliosis, highlighting the importance of individualized care in facilitating nusinersen administration. The findings reveal a high level of adherence to nusinersen treatment, even during the challenging period of the COVID-19 pandemic, underscoring the importance of nusinersen treatment for the adult SMA population. These findings contribute significantly to narrowing the knowledge gap regarding nusinersen use in an adult SMA population in China and add to the global evidence on nusinersen treatment adherence. The insights gained from this study may inform future guidelines and standardization efforts in nusinersen administration, ultimately enhancing the care provided to adults with 5q-SMA.

## Supplementary Information

Supplementary Material 1. Title of data: Hospital List. Description of data: This file provides the names of the 12 general and specialized hospitals across China that served as study sites for the multicenter registry of adults with 5q-SMA.

## Data Availability

Requests from qualified investigators for anonymized data not reported in this article should be submitted to [ https://vivli.org].

## Abbreviations

COVID-19: Coronavirus Disease 2019
CT: Computed Tomography
DMT: Disease-Modifying Therapy
EHR: Electronic Health Record
LP: Lumbar Puncture
mRNA: Messenger Ribonucleic Acid
Q1: First Quartile
Q3: Third Quartile
SD: Standard Deviation
SMA: Spinal Muscular Atrophy
SMN: Survival Motor Neuron
US: United States
