# Supplementary material for: Administration practice and adherence of nusinersen in adults with 5q-spinal muscular atrophy in China: an ambispective multicenter study
Source: BMC Neurol. 2026 Feb 28;26:176. doi: 10.1186/s12883-026-04730-x (PMC13001292; doi:10.1186/s12883-026-04730-x)
Supplement: Supplementary file 1 — Supplementary Material 1. Title of data: Hospital List. Description of data: This file provides the names of the 12 general and specialized hospitals across China that served as study sites for the multicenter registry of adults with 5q-SMA. [file 12883_2026_4730_MOESM1_ESM.docx]

# **Hospital list**

1. The First Affiliated Hospital, Sun Yat-sen University

2. Peking Union Medical College Hospital, Chinese Academy of Medical Sciences

3. Huashan Hospital, Fudan University

4. Qilu Hospital of Shandong University

5. West China Hospital, Sichuan University

6. The First People’s Hospital of Yunnan Province

7. The Second Hospital of Hebei Medical University

8. The First Affiliated Hospital of Soochow University

9. The First Affiliated Hospital of Nanchang University

10. Peking University Shenzhen Hospital

11. Xiangya Hospital of Central South University

12. The First Affiliated Hospital of Fujian Medical University
